# MicroRNAs in Osteoclastogenesis and Function: Potential Therapeutic Targets for Osteoporosis

**DOI:** 10.3390/ijms17030349

**Published:** 2016-03-09

**Authors:** Xiao Ji, Xiang Chen, Xijie Yu

**Affiliations:** Laboratory of Endocrinology and Metabolism, Department of Endocrinology, West China Hospital, Sichuan University, 610041 Chengdu, China; jixiao0321@hotmail.com (X.J.); onlycx@163.com (X.C.)

**Keywords:** microRNAs, osteoclasts, osteoclastogenesis, bone resorption, osteoporosis

## Abstract

Abnormal osteoclast formation and resorption play a fundamental role in osteoporosis pathogenesis. Over the past two decades, much progress has been made to target osteoclasts. The existing therapeutic drugs include bisphosphonates, hormone replacement therapy, selective estrogen receptor modulators, calcitonin and receptor activator of nuclear factor NF-κB ligand (RANKL) inhibitor (denosumab), *etc.* Among them, bisphosphonates are most widely used due to their low price and high efficiency in reducing the risk of fracture. However, bisphosphonates still have their limitations, such as the gastrointestinal side-effects, osteonecrosis of the jaw, and atypical subtrochanteric fracture. Based on the current situation, research for new drugs to regulate bone resorption remains relevant. MicroRNAs (miRNAs) are a new group of small, noncoding RNAs of 19–25 nucleotides, which negatively regulate gene expression after transcription. Recent studies discovered miRNAs play a considerable function in bone remodeling by regulating osteoblast and osteoclast differentiation and function. An increasing number of miRNAs have been identified to participate in osteoclast formation, differentiation, apoptosis, and resorption. miRNAs show great promise to serve as biomarkers and potential therapeutic targets for osteoporosis. In this review, we will summarize our current understanding of how miRNAs regulate osteoclastogenesis and function. We will further discuss the approach to develop drugs for osteoporosis based on these miRNA networks.

## 1. Introduction

Osteoporosis, the growing metabolic skeletal disorder worldwide, has been called a “silent killer” due to its high rate of incidence and disability. Currently, drug selection for osteoporosis is limited. Bisphosphonates, the first-line drugs for osteoporosis due to their low price and high efficiency in reducing the risk of fracture, have gastrointestinal side-effects and, more importantly, long-term application of bisphosphonates inhibit the osteoblast and osteoclast functions simultaneously [1]. Bone formation and bone resorption are elaborate and well-coupled processes. The inhibition of bone resorption will lead to inhibition of bone formation and ultimately affect the efficacy of anti-bone-resorption drugs. Previous studies suggested that, except for coupling factors, the maintenance of osteoclast numbers is very important for keeping normal osteoblast functions. For example, bisphosphonates promote osteoclast apoptosis and decrease osteoclast numbers, which lead to inhibition of osteoblast functions. On the contrary, the application of inhibitors of cathepsin K and chloride channel 7, which are all involved in bone resorption, have no effects on osteoblast functions, since these inhibitors only reduce bone resorption capacity but not osteoclast numbers. Abnormal osteoclast formation and resorption play a fundamental role in osteoporosis pathogenesis. Therefore, understanding osteoclast proliferation, differentiation, apoptosis, bone resorption, and the coupling mechanism between osteoclasts and osteoblasts, have a key role in the development of new drugs for osteoporosis.

MicroRNAs (miRNAs) are a class of small, noncoding RNAs of 19–25 nucleotides, which exist widely in eukaryotes and are highly conserved during biological evolution. After binding to 3’-untranslated regions (3’-UTR) within a target mRNA, miRNAs play a negative role in gene expression by regulating transcript localization, polyadenylation, and translation [2,3,4]. In 1993, lin-4RNAs were first discovered in *Caenorhabditis elegans* by Lee *etc.* [5]. In 2004, Chen *et al.* identified three miRNAs, which were not only specifically expressed in hematopoietic cells but the expression was dynamically modulated during early hematopoiesis and lineage commitment as well [6]. Since then, an increasing number of miRNAs have been identified to participate in osteoclast formation, differentiation, apoptosis, and resorption.

We have searched literature from PubMed and referred three other reviews [7,8,9]. This review aims to summarize our current understanding of how miRNAs regulate osteoclastogenesis and briefly refer to their potential clinical implications, such as biomarkers and the development of new drugs for osteoporosis based on these miRNA networks.

## 2. Bone Remodeling and Osteoclasts

Bone remodeling is a dynamic process throughout the whole lifetime of an individual, by which the skeleton maintains its structural integrity and exerts its metabolic functions as a repository of calcium and phosphorus [10]. Bone remodeling is regulated by the subtle equilibrium between osteoblastic bone formation and osteoclastic bone resorption. Firstly, there is an “activation” phase and a “resorption” phase. Cytokines are released at the site of remodeling to recruit osteoclasts to the bone surface. These osteoclasts form a ruffled border allowing them to adhere to the bone surface tightly. Between the osteoclast and the underlying bone, there exists a tiny isolated microenvironment into which the osteoclast’s proton pump releases ions that create an acidic environment, making the mineralized component of the bone matrix dissolve. The organic matrix is exposed and degraded by cathepsin K [11]. Subsequently, the “reversal” phase begins. Mononuclear cells prepare the bone surface for osteoblasts and provide signals to recruit them. Along with proliferating, early osteoblasts secrete an extracellular matrix, which contains type I collagen abundantly. This matrix matures and is mineralized, and osteoblasts continue to differentiate. Finally, the bone surface is repaired. Those mature osteoblasts either undergo apoptosis, or eventually differentiate into osteocytes or bone surface lining cells [12].

Originating from mononuclear hematopoietic myeloid lineage cells, osteoclast precursors (OCPs) are formed in the bone marrow and subsequently attracted to the bloodstream by chemokines. Attracted by a variety of factors released from bone remodeling units (BRUs), OCPs are attracted back into bones and then they differentiate into osteoclasts [13]. During normal physiological conditions, osteoclastogenesis is regulated by osteoblasts and stromal cells, both of which provide two essential factors, macrophage colony-stimulating factor (M-CSF) and receptor activator of nuclear factor NF-κB ligand (RANKL). M-CSF plays an essential role in the survival, proliferation and the expression of RANK in early OCPs (monocyte/macrophage lineage). Therefore, the primary role of M-CSF is to provide survival signals during osteoclastogenesis [14]. In contrast, RANKL provides osteoclast differentiation signals and activates multiple signal transduction pathways, which turn on transcription factors NF-κB, c-Fos, transcription factor nuclear factor of activated T cells (NFATc1), and microphthalmia-induced transcription factor (MITF) [15].

## 3. Signaling Pathways of Osteoclast Differentiation

**Figure 1:**
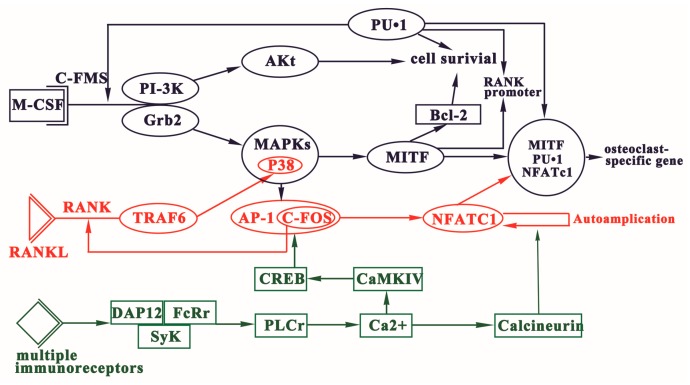
Three signaling pathways of osteoclast differentiation. Osteoclastogenesis is essentially modulated by three signaling pathways activated by M-CSF, RANKL, and ITAM. The binding of M-CSF to its receptor c-Fms play function in the survival of osteoclast precursor cells. RANKL-RANK signaling commits the osteoclast precursors (OCPs) to osteoclast fate. ITAM-dependent co-stimulatory signals are imperative to RANKL-RANK signaling.

Osteoclast differentiation is essentially modulated by three signaling pathways, which are activated by macrophage colony-stimulating factor (M-CSF), receptor activator of nuclear factor NF-κB ligand (RANKL), and immunoreceptor tyrosine-based activation motif (ITAM), respectively (Figure 1).

### 3.1. Regulation of OCPs Formation via M-CSF Signaling

M-CSF is pivotal for the survival and proliferation of OCPs. M-CSF, through its receptor c-Fms, transmits signals to the cell and then activates extracellular signal-regulated kinase (ERK) through Grb2 and phosphoinositide 3-kinase (PI-3K)/Akt [16,17]. The differentiation process of hematopoietic stem cells to OCPs is induced by transcription factors, such as purine-rich binding protein 1 (gene symbol: SPI1, PU.1) and Mitf [18]. PU.1 is a hematopoietic-specific member of the ETS family [19]. Deletion of PU.1 in mice results in a complete lack of OCPs leading to osteopetrosis [20]. There are PU.1 binding sites within promoters of many genes involved in osteoclast formation and function [21]. During the process of hematopoietic stem cells differentiating into the monocyte/macrophage lineage, PU.1 stimulates the expression of CSF1R which is the receptor of CSF1 (*i.e.*, M-CSF) [22]. By upregulating the transcription factor c-FOS, CSF1R induces expression of receptor activator of NFκB (RANK; TNFRSF11A). In cooperation with other transcription factors, PU.1 modulates the RANK gene transcription [23]. Activator protein 1 (AP-1) plays a critical role in osteoclastogenesis. The AP-1 transcription factor complexes comprise of the Fra, Fos, Jun, and activating transcription factor (ATF) families.

MITF is another critical transcription factor participating in the late stages of osteoclastogenesis. Through a conserved mitogen-activated protein kinase (MAPK) consensus site, M-CSF induces phosphorylation of MITF [24]. Then MITF induces the expression of BCL-2 and promotes macrophage survival. Both Mitf^mi/mi^ and Bcl2^−/−^ mice suffer severe osteopetrosis [25]. Moreover, through binding to the sites within the RANK promoter, MITF and PU.1 increase RANK promoter activity three-fold and two-fold, respectively, and six-fold synergistically [26]. Conversely, Mitf-E levels are significantly upregulated by RANKL [27].

Both PU.1 and MITF not only play an important role in the survival of OCPs, but also participate in osteoclast-specific gene induction at the terminal stage of differentiation [21,28].

### 3.2. RANKL-RANK Signaling

RANKL commits the OCPs to osteoclast fate. The activation of RANKL-RANK signaling leads to the expression of genes involving the fusion of mononuclear osteoclast precursors, like dendritic cell-specific transmembrane protein (DC-STAMP), as well as of genes regulating resorption capacity of multinucleated osteoclasts, including cathepsin K, chloride channel 7, matrix metalloprotein 9, and calcitonin receptor.

RANKL-RANK binding recruits TRAF-6 to activate PI-3K, NF-κB family of transcription factors and all three MAPK pathways, including ERK, JNK (Janus N-terminal kinase), and p38. NF-κB is required for the expression of a variety of cytokines, including IL-6, IL-1, TNF-α, GM-CSF, RANKL, and other growth factors. Protein kinase p38 is activated via phosphorylation of MAPK kinase (MKK) 6. The activation of p38 results in the downstream activation of MITF [29]. Hence, MITF exists downstream of the M-CSF and RANKL signaling pathways. Treatment with the p38 inhibitors increases phosphorylation of ERK, showing a balance between ERK and p38 phosphorylation.

RANKL induces the expression of the AP-1 complexity, which consists of Fos (c-Fos, FosB, Fra-1, Fra-2) and Jun (c-Jun, JunB, JunD) [30]. NFATc1 expression is dependent on the TRAF-6-NF-κB and c-Fos pathways, which are activated by both RANKL and Ca^2+^ signaling. NFATc1 regulates OC-specific genes, such as tartrate resistant acid phosphatase (TRAP) [31], Cathepsin K [21], calcitonin receptor, osteoclast-associated receptor (OSCAR) [32], and β3 integrin [33].

### 3.3. Immunoreceptor Tyrosine-Based Activation Motif (ITAM)-Dependent Costimulatory Signals

M-CSF and RANKL are not sufficient to activate the signals required for osteoclastogenesis. Immunoreceptor tyrosine-based activation motif (ITAM)-dependent costimulatory signals, activated by multiple immunoreceptors, are essential for osteoclastogenesis. Both Fc receptor common γ subunit (FcRγ) and DNAX-activating protein 12 (DAP12) are ITAM-harboring adapters. In osteoclast precursor cells, FcR and DAP12 associated with multiple immunoreceptors activate calcium signaling through phospholipase C [34]. These receptors include OSCAR, triggering receptor expressed in myeloid cells-2 (TREM-2), signal-regulatory protein β1 (SIRPβ1), and paired Ig-like receptor-A (PIR-A). These receptor-mediated signals cannot substitute RANKL but act with RANKL cooperatively. Therefore, ITAM-mediated signals can be identified as co-stimulatory signals for RANK.

## 4. miRNAs in Osteoclasts

**Figure 2:**
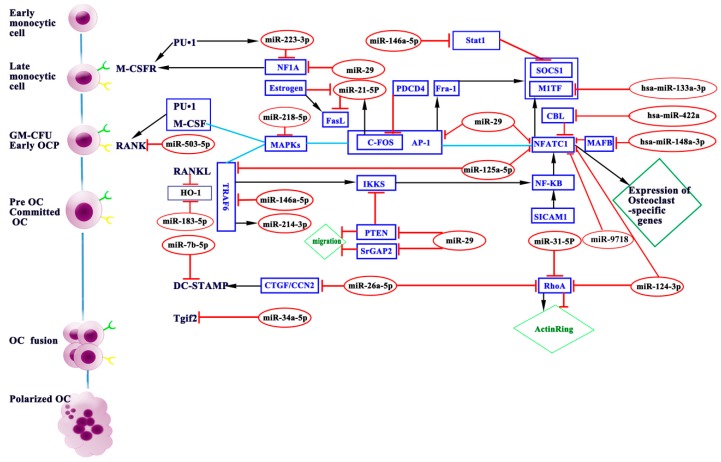
miRNAs modulate osteoclast differentiation and function. Through repressing varieties of target genes, miRNAs participate in osteoclast differentiation and maturation. Blue boxes indicate targets of miRNAs. Red ovals indicate miRNAs involved in osteoclastogenesis. Green receptors indicate M-CSFR, and yellow receptors indicate RANK.

Osteoclast differentiation is regulated by transcriptional, post-transcriptional, and post-translational mechanisms. miRNAs are fundamental post-transcriptional regulators of gene expression. miRNAs play a key role in the normal bone development. Heterozygous microdeletions in the MIR17HG locus, encoding microRNA 17–92 cluster, lead to autosomal dominant Feingold syndrome in humans, characterized by short stature, microcephaly, and abnormal development of fingers and toes [35]. Further study on animal models carrying targeted deletions of individual components of miR-17~92 revealed that miR-17 seed family is critical in patterning of the axial skeleton [36]. MicroRNA-related single nucleotide polymorphisms (SNPs) also have a potential impact on the skeletal phenotype [37]. Furthermore, emerging evidence suggests that miRNAs are involved in the multiple biological and pathological processes in osteoclast proliferation, differentiation, apoptosis, cytoskeleton formation, and bone resorption. During the early, middle, and late stages of murine osteoclastogenesis, miRNA microarray analysis showed 49 miRNAs were upregulated and 44 were downregulated [38]. In the following section, we will discuss relevant miRNAs in osteoclasts and their potential targets and signaling pathways (Table 1 and Figure 2).

### 4.1. miRNAs Promoting Osteoclastogenesis

#### 4.1.1. miR-21-5p

miR-21-5p is highly expressed in osteoclast precursors, and its expression levels are upregulated during RANKL-induced osteoclastogenesis [39]. miR-21-5p simultaneously promotes osteoclastogenesis and induces osteoclastic apoptosis. Transcription factors for osteoclastogenesis, such as c-Fos and PU.1, trigger miR-21-5p transcription via binding to the promoter of miR-21-5p [40]. miR-21-5p downregulates programmed cell death 4 (PDCD4) protein levels and, thus, removes its inhibitory effect on c-Fos. At the same time, RANKL-induced c-Fos upregulates miR-21-5p expression. Therefore, c-Fos/miR-21-5p/PDCD4 forms a positive feedback loop, promoting RANKL-induced osteoclastogenesis [39]. In addition, miR-21-5p plays a critical role in estrogen-related osteoclastogenesis. Estrogen inhibits osteoclastogenesis and induces osteoclastic apoptosis [41]. Estrogen attenuates miR-21-5p biogenesis; therefore, the protein levels of FasL (Fas ligand), a target of miR-21-5p, are post-transcriptionally increased, which induces osteoclastic apoptosis [42].

#### 4.1.2. miR-29

miR-29 family includes miR-29a-3p, miR-29b-3p, and miR-29c-3p. During osteoclastogenesis from bone marrow monocytes (BMMs) and monocytic cell line RAW264.7, all miR-29 family members increased, which was consistent with the expressions of osteoclast markers TRAP and cathepsin K [43]. Knockdown of miR-29 inhibited commitment and migration of pre-osteoclasts without interfering cell viability, actin ring formation, or apoptosis in mature osteoclasts [43]. The target genes of miR-29 family are Calcr (calcitonin receptor), RNAs critical for cytoskeletal organization, including Cdc42 (cell division control protein 42) and Srgap2 (SLIT-ROBO Rho GTPase-activating protein 2), and those associated with the macrophage lineage, including Gpr85 (G protein-coupled receptor 85), Nfia (nuclearfactor I/A), and Cd93 [43].

#### 4.1.3. miR-31-5p

miR-31-5p was highly upregulated during osteoclastogenesis under RANKL induction [44]. Inhibition of miR-31-5p attenuated the RANKL-induced osteoclast formation and bone resorption [44]. Further study suggested that actin ring formation in osteoclasts was severely impaired by miR-31-5p inhibition. RhoA, a target gene of miR-31-5p, was upregulated by miR-31-5p inhibition [44]. RhoA plays a key role in actin ring formation in osteoclasts. Therefore, miR-31-5p is a positive regulator of osteoclast formation and bone resorption through targeting RhoA.

#### 4.1.4. Hsa-miR-133a-3p and Hsa-miR-422a

The expression of hsa-miR-133a-3p and hsa-miR-422a was upregulated in circulating monocytes of low bone mineral density (BMD) post-menopausal Caucasian women [46,47]. Bioinformatic analysis revealed that three osteoclast-related genes to be target for hsa-miR-133a-3p [45], including CXCL11, CXCR3, and SLC39A1; however, the negative correlations between hsa-miR-133a-3p and all three genes had no statistical significance using Pearson correlation analysis [45]. Bioinformatic analysis also predicted several potential target genes for hsa-miR-422a [46]. Negative correlations between hsa-miR-422a and five of these genes (CBL, CD226, IGF1, PAG1, and TOB2) had statistical significance [46]. Therefore, both hsa-miR-133a-3p and hsa-miR-422a may act as potential microRNA biomarkers for post-menopausal osteoporosis.

#### 4.1.5. Hsa-miR-148a-3p

miRNA expression profile analysis showed miR-148a-3p was dramatically upregulated during osteoclastogenesis from human CD14+ peripheral blood mononuclear cells (PBMCs) using microarray [47]. Overexpression of hsa-miR-148a-3p in CD14+ PBMCs facilitated osteoclastogenesis, whereas inhibition of hsa-miR-148a-3p depressed it [47]. V-maf musculoaponeurotic fibrosarcoma oncogene homolog B (MAFB) is a target of hsa-miR-148a-3p, which negatively regulates RANKL-induced osteoclastogenesis [47]. Therefore, hsa-miR-148a-3p acts as a positive regulator of osteoclastogenesis through inhibiting MAFB. The higher expression level of hsa-miR-148a-3p in CD14+ PBMCs from lupus patients may play a role in the enhanced osteoclastogenesis and lower BMD [47,48].

#### 4.1.6. miR-183-5p

miR-183-5p is a member of miR-182-183 cluster located on the 7q31-34. This cluster includes miR-96, miR-182 and miR-183 [49]. miR-183-5p expression is upregulated during RANKL-induced osteoclastogenesis from BMMs. Inhibition of miR-183-5p led to a substantial reduction in TRAP positive multinucleated OCs [49]. Heme oxygenase-1 (HO-1) is predicted to be a target of miR-183-5p. RANKL decreased the expression of HO-1 and stimulated osteoclastogenesis. miR-183-5p inhibitor increased the expression of HO-1 and decreases osteoclast differentiation [49]. Therefore, miR-183-5p is a positive regulator of osteoclastogenesis through inhibiting HO-1 expression.

#### 4.1.7. miR-214-3p

miR-214-3p is highly conserved across vertebrates and has been suggested to play an important role in vertebrate skeletal development [50]. miR-214-3p is upregulated during osteoclastogenesis from BMMs induced by M-CSF and RANKL [51]. Overexpression of miR-214-3p in BMMs facilitates osteoclastogenesis, whereas inhibition of miR-214-3p depresses it [51]. Phosphatase and tensin homolog (PTEN), a tumor suppressor, negatively regulates PI3K/Akt signaling as a lipid phosphatase [52]. PTEN plays an essential role in regulating cell proliferation, migration, invasion, and apoptosis [52]. Phosphorylated PTEN reduces osteoclastogenesis by inhibiting the PI3K/Akt pathway [53]. *Zhao et al*. suggested that miR-214-3p regulates osteoclastogenesis through targeting the PTEN/PI3k/Akt pathway [51].

#### 4.1.8. miR-223-3p

miR-223-3p has been well-studied in osteoclastogenesis. Like miR-21-5p, miR-223-3p is downregulated during osteoclast differentiation [54]. There are conflicting studies indicating that miR-223-3p is capable of enhancing or suppressing osteoclast differentiation [55]. Antisense to miRNA-223 significantly suppressed osteoclast differentiation and osteoclast bone resorbing activity [56]. However, overexpression of pre-miR-223 also completely blocked TRAP-positive osteoclast formation, indicating that appropriate miRNA-223 expression levels should be kept during osteoclastogenesis [57].

miR-223-3p is specifically expressed in CD11b positive myeloid cell lineages [6]. By inhibiting NFIA (transcriptional repressor Nuclear Factor IA) expression, miR-223-3p regulates osteoclast differentiation. NFIA, a CCAAT-box binding transcription factor, is a member of the dimeric DNA-binding nuclear factors I (NFI) protein family [58]. miR-223-3p binds to specific sites within the promoter of NFIA and represses transcription by influencing epigenetic events [59]. In turn, NFIA and C/EBPα compete for binding to the miR-223-3p promoter, maintaining miR-223-3p expression levels [60]. When miR-223-3p expression is extremely low, NFIA will be upregulated, thus blocking osteoclast differentiation.

PU.1, miR-223-3p, NFIA and macrophage colony-stimulating factor receptor (M-CSFR) form a positive feedback loop. PU.1 upregulates miRNA-223 expression and then miRNA-223 downregulates NFIA expression, resulting in upregulation of M-CSFR [56]. In conflict with the above PU.1/miR-223-3p/NFIA positive feedback theory, miR-223-3p overexpression was reported to block osteoclast differentiation in RAW264.7 cells [57] and peripheral blood mononuclear cells (PBMCs) [62]. IKKα, a critical factor in the NF-κB pathway, is downregulated by miR-223-3p for inhibiting differentiation of osteoclasts [7]. Through excess p52 in the absence of RelB, the non-canonical NF-κB pathway involved in IKKα, could be one explanation for the dual role of miR-223-3p in osteoclast differentiation [61].

#### 4.1.9. miR-9718

miR-9718 is highly conserved between human and mouse, which is preferentially expressed in bone tissue, especially in osteoclasts [63]. The expression level of miR-9718 significantly increased during osteoclastogenesis from BMMs and RAW264.7 cells. Upregulation of miR-9718 in RAW264.7 cells facilitated M-CSF and RANKL-induced osteoclastogenesis, whereas inhibition of miR-9718 repressed it [63]. PIAS3 is a target of miR-9718 [63]. PIAS3 belongs to protein inhibitor of activated STAT (PIAS) family, which consists of PIAS1, PIAS3, PIASx, and PIASy. PIAS3 inhibits osteoclastogenesis through downregulating NFATc1 and OSCAR [63]. Therefore, miR-9718 acts as a positive regulator of osteoclastogenesis by targeting PIAS3.

### 4.2. miRNAs Inhibiting Osteoclastogenesis

#### 4.2.1. miR-7b-5p

miR-7b-5p was markedly downregulated during osteoclastogenesis from RAW264.7 cells induced by M-CSF and RANKL. Overexpression of miR-7b-5p in RAW264.7 cells decreased the numbers of TRAP-positive multinucleated cells, whereas inhibition of miR-7b-5p enhanced osteoclastogenesis [64]. DC-STAMP is predicted to be a target of miR-7b-5p. DC-STAMP is a key regulator of cell-cell fusion for the formation of mature osteoclast. Therefore, miR-7b-5p is a negative regulator of osteoclastogenesis through inhibiting DC-STAMP and its downstream signal factors including NFATc1, c-Fos, Akt, Irf8, Mapk1, and Traf6 [64].

#### 4.2.2. miR-26a-5p

miR-26a-5p is an important regulator of cell proliferation and differentiation. The miR-26a-5p expression was upregulated at the late stage of osteoclastogenesis induced by RANKL [65]. Expression of an miR-26a-5p mimic in osteoclast precursor cells attenuated osteoclastogenesis by suppressing the expression of connective tissue growth factor/CCN family 2 (CTGF/CCN2), while inhibitor of miR-26a-5p enhanced RANKL-induced osteoclastogenesis as well as CTGF expression [65]. CTGF can promote osteoclastogenesis via upregulation of DC-STAMP. Therefore, miR-26a-5p is a negative regulator of osteoclastogenesis by targeting CTGF [65].

#### 4.2.3. miR-34a-5p

miR-34a-5p is highly conserved between mouse and human. The expression of miR-34a-5p is downregulated during osteoclast differentiation [66]. One miR-34a-5p precursor (pre-miR-34a) significantly depressed osteoclast differentiation both from mouse BMMs and from PBMCs [66]. Osteoclastic miR-34a-5p-overexpressing transgenic mice showed lower bone resorption and higher bone mass. On the contrary, miR-34a-5p knockout and heterozygous mice exhibited augmented bone resorption and lower bone mass [66]. Mechanistically, transforming growth factor-b-induced factor 2 (Tgif2), a novel regulator of osteoclastogenesis by stimulating the activity of NFATc1, NF-κB, and c-Jun, has been demonstrated to be a target of miR-34a-5p [66]. Therefore, miR-34a-5p serves as a negative regulator of osteoclastogenesis by inhibiting Tgif2.

#### 4.2.4. miR-124-3p

miR-124-3p has been suggested to have a putative tumor-suppressive role. Previous study indicated it also may be an intrinsic negative regulator of osteoclast differentiation by suppressing NFATc1 expression. NFATc1 is a key regulator of osteoclastogenesis. TargetScan, a web-based bioinformatics tool, predicted two conserved binding sequences of miR-124-3p in the 3′UTR region of mouse NFATc1 gene. During the osteoclastic differentiation from BMMs induced by RANKL, the expression of miR-124-3p rapidly decreased. Pre-miR-124 significantly inhibited the RANKL-induced NFATc1 induction and osteoclast differentiation. On the contrary, inhibition of miR-124-3p potently promoted NFATc1 expression and osteoclastogenesis. miR-124-3p also reduces the expression of RhoA and Rac1, through which it might inhibit the migration of osteoclast precursors [67].

#### 4.2.5. miR-125a-5p

miR-125a-5p played a negative function in osteoclastogenesis through a novel TRAF6/NFATc1/miR-125a-5p regulatory feedback loop [68]. During M-CSF and RANKL-induced osteoclastogenesis from circulating CD14+ PBMCs, miR-125a-5p was dramatically downregulated [68]. Overexpression of miR-125a-5p in CD14+ PBMCs inhibited osteoclastogenesis, and *vice versa* [68]. Overexpression of NFATc1 inhibited miR-125a-5p transcription [68].

#### 4.2.6. miR-146a-5p

miR-146a-5p, a negative regulator of immune and inflammatory responses, is upregulated in rheumatoid arthritis (RA) synovium and PBMCs [70]. miR-146a-5p directly targets signal transducer and activator transcription 1 (Stat1), and Stat1 selectively attenuates of SOCS1 [71]. Yao *et al.* successfully transported miR-146a into human PBMCs and subsequently demonstrated the inhibitory function of miR-146a in osteoclastogenesis [69].

#### 4.2.7. miR-218-5p

miR-218-5p has been demonstrated to stimulate bone formation. miR-218-5p has also been suggested to be a negative regulator of osteoclastogenesis. The expression of miR-218-5p was decreased in CD14+ PBMCs from post-menopausal osteoporosis patients compared with healthy control. During osteoclastogenesis from BMMs and RAW264.7 induced by RANKL, miR-218-5p expression was significantly downregulated. Upregulation of miR-218-5p obviously inhibited the formation of multinuclear osteoclasts, the migration of osteoclast precursors, actin ring formation, and bone resorption along with the decreased TRAP and Cathepsin K expression. Mechanistically, miR-218-5p suppresses osteoclastogenesis by targeting the p38MAPK-c-Fos-NFATc1 pathway [72].

#### 4.2.8. miR-503-5p

miR-503-5p has also been found to be downregulated in CD14+ PBMCs from post-menopausal osteoporosis patients compared with healthy controls [73]. miR-503-5p, located on Xq26.3, facilitates monocytic differentiation by targeting cell-cycle regulators. Overexpression of miR-503-5p in CD14+ PBMCs inhibited RANKL-induced osteoclastogenesis. Conversely, silencing miR-503-5p in CD14+ PBMCs promoted osteoclastogenesis [73]. Mechanistically, miR-503-5p acts as a negative regulator of osteoclastogenesis through inhibiting RANK, which was confirmed to be a target of miR-503-5p [73].

## 5. Potential Clinical Implications of miRNAs and miRNAs-Based Therapeutic Strategy for Osteoporosis

The existing therapeutic drugs include bisphosphonates, hormone replacement therapy, selective estrogen receptor modulators, calcitonin, and RANK ligand inhibitor (denosumab), *etc.* Bisphosphonates are nowadays the first-line anti-resorptive medication. There exist several potential adverse clinical events concomitant with the medication of bisphosphonates, including osteonecrosis of the jaw [74], atrial fibrillation [75], acute inflammatory response [75], and oversuppression of bone turnover [76]. Bone formation and bone resorption are well-coupled processes. The inhibition of bone resorption will result in inhibition of bone formation [1].

miRNAs are involved in the osteoclast proliferation, differentiation, cell-fusion, apoptosis, cytoskeleton formation, and bone resorption, which were summarized in Table 1 and Figure 2. As stated above, most miRNAs are involved in promoting or inhibiting osteoclast formation and maturation. Only a few miRNAs are capable of affecting osteoclast function.

### 5.1. Potential Use of miRNAs as Biomarkers

The role of miRNAs as biomarkers for bone diseases has drawn much attention recently.

#### 5.1.1. Biomarkers for Osteoclasts Activity

As a positive regulator in osteoclast formation, the expression of miR-29 (a/b/c) family was upregulated during osteoclastogenesis *in vitro* cell culture, which was consistent with the expression of osteoclast markers TRAP and cathepsin K [43]. In contrast, upregulation of miR-218-5p, a negative ocstoclastogenesis regulator, was consistent with the decreased TRAP and Cathepsin K expression [72]. The expression of plasma TRAP5b has been used to estimate the activity of osteoclasts; thus, miR-29 and miR-218-5p need further study to validate their potential as novel biomarkers for osteoclasts activity.

Panache *et al.* discovered miR-21-5p expression was correlated with bone resorption marker CTX [77].

#### 5.1.2. Biomarkers for Post-Menopausal Osteoporosis

Both miR-133a-3p and miR-422a were upregulated in human circulating monocytes from the lower BMD post-menopausal Caucasian women groups [45,46]. In contrast, the expression of miR-218-5p and miR-503-5p was decreased in CD14+ PBMCs from post-menopausal osteoporosis patients compared with healthy controls [72,73]. Therefore, miR-133a-3p, miR-422a, miR-218-5p, and miR-503-5p have potential acting as biomarkers for post-menopausal osteoporosis.

Panache *et al.* discovered miR-122-5p, miR-125b-5p, and miR-21-5p were upregulated in serum from osteoporotic fracture patients compared with osteoarthritic controls [77]. Li *et al.* have validated downregulation of miR-21-5p and upregulation of miR-133a-3p in the plasma from osteoporosis and osteopenia patients *versus* normal Chinese post-menopausal women. Additionally, the circulating expression levels of miR-21-5p and miR-133a-3p were found to be correlated with the BMD [78].

Seeliger *et al.* have identified 11 miRNAs, including miR-21-5p, miR-23-3p, miR-24-3p, miR-25-3p, miR-27a-3p, miR-100-5p, miR-122a-5p, miR-124-3p, miR-125b-5p, miR-148a-3p, and miR-223-3p, were significantly upregulated in the serum and plasma from osteoporotic fracture patients compared with the nonosteoporotic patients’ samples through a miRNA array. Except miR-25-3p, miR-27a-3p and miR-223-3p, the other nine miRNAs were subsequently validated their upregulation in the serum of patients with osteoporosis. However, only six miRNAs, miR-21-5p, miR-23-3p, miR-24-3p, miR-25-3p, miR-100-5p, and miR-125b-5p, exhibited a significant upregulation in bone tissue of osteoporotic patients. In total, five miRNAs showed an upregulation both in serum and bone tissue and these five miRNAs have potential acting as biomarkers for osteoporotic diagnosis [79].

Weilner *et al.* discovered three downregulated miRNAs (miR 133b, miR-328-3p, let-7g-5p) and three upregulated miRNAs (miR-10a-5p, miR-10b-5p, miR-22-3p) in serum from post-menopausal women who recently suffered osteoporotic fractures at the femoral neck relative to control samples. miR-328-3p and let-7g-5p were validated downregulators, subsequently, while miR-22-3p was identified as a significant downregulator in the validation cohort. Moreover, let-7g-5p, miR-10b-5p, miR-100-5p, miR-148a-3p, and miR-21-5p were found to impact osteogenesis through osteogenic differentiation of human mesenchymal stem cells (MSCs) *in vitro* [80]. Garmilla *et al.* found miR-187-3p, and miR-518f-3p differentially regulated in osteoporotic bone [81]. Meng *et al.* found six miRNAs (miR-130b-3p, miR-151a-3p, miR-151b, miR-194-5p, miR-590-5p, and miR-660-5p) were significantly upregulated in the blood of 48 post-menopausal Chinese participants with osteoporosis compared to those with osteopenia. Except miR-660-5p, the other five miRNAs were confirmed the increased expression through external validation in 24 additional post-menopausal women. Moreover, high miRNA expression levels of miR-130b-3p, miR-151a-3p, miR-151b, and miR-194-5p were identified to be significantly correlated with low BMD levels [82].

Taken together, the role of miRNAs as osteoporotic biomarkers mostly attributes to a causal relationship between the miRNA and the osteoblast differentiation [80]. Interestingly, miRNAs, like miR-148a-3p [47] and miR-21-5p [39,40,41,42], were found to be involved both in osteogenesis and the regulation of osteoclastogenesis. Further cross validation of cell free blood-based miRNAs with bone miRNAs is strongly relevant.

### 5.2. Potential of miRNAs as Therapeutic Targets in Osteoporosis

#### 5.2.1. Transgenic Mice

Osteoclastic miR-34a-5p-overexpressing transgenic mice showed lower bone resorption and higher bone mass. On the contrary, miR-34a-5p knockout and heterozygous mice exhibited augmented bone resorption and lower bone mass [66].

#### 5.2.2. miRNA Delivery System

Through a novel miRNA delivery system based on bacteriophage MS2 virus-like particles (MS2 VLPs), Yao *et al.* successfully transported miR-146a into human PBMCs, and subsequently demonstrated the inhibitory function of miR-146a-5p in osteoclastogenesis [69]. miR-148a-3p exerts potent inhibitory effects on osteoclast differentiation. Cheng *et al.* validated the bone mass in mice would increase via a single tail vein injection of a specific antagomiR-148a [47]. Liu *et al.* established efficient delivery systems to facilitate antagomir-148a-3p to bone resorption surfaces to reduce bone resorption with minimal off-target effects [48]. miR-503-5p acts as a negative regulator of osteoclastogenesis through inhibiting RANK. Ovariectomy (OVX) mice exhibited increased RANK protein expression, promoted bone resorption, and decreased bone mass after using a specific antagomir to silence miR-503-5p expression, whereas agomir-503 exhibit opposite effects [73].

In developing miRNAs-based therapy, it may be helpful to maintain the normal function of osteoblasts if only inhibiting the bone resorption function of osteoclast without affecting the number of osteoclasts. Therefore, it seems that miR-31-5p will be an ideal target, since it just promotes osteoclast function through inhibiting RhoA and RhoA plays a key role in actin ring formation as a small GTPase [44].

## 6. Conclusions

Cumulating evidence suggested that miRNAs are involved in multiple physiological and pathological processes of osteoclast differentiation and function. In general, the expression levels of miRNAs that exert inhibitory effects on osteoclastogenesis tend to decrease during osteoclast formation, and *vice versa*. MiRNAs-based therapy has been considered as a promising strategy for the treatment of osteoporosis.

## Figures and Tables

**Table 1:** Summary of miRNAs, their target genes, expression profile, and effects on osteoclasts.

miRNAs	Sample Resources	Target Gene (s)	Expression	Function	References
miR-21-5p	BMMs,c-Fos^−/−^mice, CAG-Z-miR-21-EGFP transgenic mice	FasL, PDCD4	↑	Inhibits/promotes	[39,40,41,42]
miR-29	BMMs, RAW264.7	CDC42, SRGAP2, NFIA, CD93, CALCR	↑	promotes	[43]
miR-31-5p	BMMs	RhoA	↑	promotes	[44]
hsa-miR-133a-3p	low BMD postmenopausal Caucasian women	CXCL11, CXCR3 and SLC39A1	↑	Inhibit/promotes	[45]
hsa-miR-422a	low BMD postmenopausal Caucasian women	CBL, CD226, IGF1, PAG1, TOB2	↑	promotes	[46]
hsa-miR-148a-3p	PBMCs	MAFB	↑	promotes	[47,48]
miR-183-5p	BMMs	HO-1	↑	promotes	[49]
miR-214-3p	BMMs, RAW264.7, OC-214Tg mice	Pten	↑	promotes	[50,51,52,53]
miR-223-3p	RAW264.7 cells, PBMCs, RA/OA	NFIA, IKKα	↓	Inhibits/promotes	[54,55,56,57,58,59,60,61,62]
miR-9718	RAW264.7, BMMs	PIAS3	↑	promotes	[63]
miR-7b-5p	BMMs, RAW264.7	DC-STAMP	↓	Inhibits	[64]
miR-26a-5p	BMMs	CTGF	↑	Inhibits	[65]
miR-34a-5p	BMMs, PBMCs, Tie2-cre mice, 34a-KO/Het mice, OVX mice, 34a-Tg mice	Tgif2	↓	Inhibits	[66]
miR-124-3p	BMMs	NFATc1, RhoA, Rac1	↓	Inhibits	[67]
miR-125a-5p	PBMCs	TRAF6	↓	Inhibits	[68]
miR-146a-5p	PBMCs	TRAF6, Stat1	↓	Inhibits	[69,70,71]
miR-218-5p	BMMs, RAW264.7, PBMCs	p38MAPK-c-Fos-NFATc1	↓	Inhibits	[72]
miR-503-5p	PBMCs	RANK	↓	Inhibits	[73]

↑ means miRNAs are upregulated; ↓ means miRNAs are down-regulate.

## Abbreviations

miRNAs	MicroRNAs
3’-UTR	3’-Untranslated Regions
OCPs	osteoclast precursors
BRUs	bone remodeling units
M-CSF	macrophage colony-stimulating factor
RANKL	receptor activator of nuclear factor NF-κB ligand
NFATc1	transcription factor nuclear factor of activated T cells
MITF	microphthalmia-induced transcription factor
ERK	extracellular signal-regulated kinase
PI-3K	phosphoinositide 3-kinase
PU.1	purine-rich binding protein 1
RANK	receptor activator of NFκB
AP-1	Activator protein 1
ATF	activating transcription factor
MAPK	mitogen-activated protein kinase
DC-STAMP	dendritic cell specific transmembrane protein
JNK	Janus N-terminal kinase
TRAP	tartrate resistant acid phosphatase
OSCAR	osteoclast-associated receptor
ITAM	Immunoreceptor tyrosine-based activation motif
FcRγ	Fc receptor common γ subunit
DAP12	DNAX-activating protein 12
TREM-2	triggering receptor expressed in myeloid cells-2
SIRPβ1	signal-regulatory protein β1
PIR-A	paired Ig-like receptor-A
SNPs	single nucleotide polymorphisms
PDCD4	programmed cell death 4
FasL	Fas ligand
BMMs	bone marrow monocytes
Calcr	calcitonin receptor
Cdc42	cell division control protein 42
Srgap2	SLIT-ROBO Rho GTPase-activating protein 2
Gpr85	G protein-coupled receptor 85
BMD	bone mineral density
HO-1	Heme oxygenase-1
PTEN	Phosphatase and tensin homolog
PBMCs	peripheral blood mononuclear cells
PIAS	protein inhibitor of activated STAT
CTGF/CCN2	connective tissue growth factor/CCN family 2
Tgif2	transforming growth factor-b-induced factor 2
RA	rheumatoid arthritis
Stat1	signal transducer and activator transcription 1
OVX	Ovariectomy
